# Clinicopathological Features and Prognosis Analysis of Primary Bile Duct and Ampullary Neuroendocrine Neoplasms: A Population-Based Study from 1975 to 2016

**DOI:** 10.3390/curroncol30010036

**Published:** 2022-12-29

**Authors:** Yijun Li, Rui Hua, Jianjun He, Huimin Zhang

**Affiliations:** 1 Department of Breast Surgery, The First Affiliated Hospital of Xi’an Jiaotong University, Xi’an 710061, China; 2 Department of Cardiovascular Medicine, The First Affiliated Hospital of Xi’an Jiaotong University, Xi’an 710061, China

**Keywords:** neuroendocrine neoplasms, SEER database, survival analysis, nomogram, bile duct

## Abstract

Background: The main purpose of this study is to analyze the clinicopathological features and prognosis factors of bile duct and ampullary neuroendocrine neoplasms (NENs). Methods: The relevant data were collected from the SEER database from 1975 to 2016. The Kaplan–Meier curve and Cox model were used for survival analysis. The nomogram was drawn to predict the survival rate. The calibration, discrimination and clinical utility of the nomogram were evaluated by calibration curve, the concordance index (C-index) and decision curve analysis (DCA). Results: A total of 340 cases were included in our research. According to Kaplan–Meier analysis, 1-year, 3-year and 5-year of overall survival (OS) were 77.3%, 61.9% and 58.4%, while 1-year, 3-year and 5-year of the disease-specific survival (DSS) were 82.7%, 69.3% and 66.9%, respectively. The multivariable analysis results showed that age, histological grade, SEER stage and surgery were independent predictors for either OS or DSS. The calibration curve and the C-index value indicated that the nomogram was well calibrated and had good discrimination. DCA showed that the model had ideal net benefits. Conclusions: The age, histological grade, SEER stage and surgery were identified as independent prognostic variables for OS and DSS. After verification, nomogram has good predictive ability and clinical application value.

## 1. Introduction

Neuroendocrine neoplasms (NENs) are a class of tumors derived from peptidergic neurons and neuroendocrine cells, most often occurring in the gastrointestinal tract and pancreatic tissue [1]. Due to the lack of neuroendocrine cells, NENs are rare in the biliary tract, accounting for only 0.2–2.0% of biliary tract tumors [2]. The most common sites of bile duct NENs were the common hepatic duct and the terminal segment of the common bile duct (19.2%), followed by the common bile duct (17.9%) [3,4]. The etiology of NENs is still unclear. Some studies have shown that NENs were related to chronic inflammation caused by cholelithiasis and congenital biliary malformation. Long-term chronic inflammation leads to the gradual metaplasia of bile duct epithelial cells into NENs [5].

According to whether there is hormone secretion or not and the related manifestations of excessive secretion, NENs are divided into functional NENs and non-functional NENs. Due to the role of hormones, the former often presents typical symptoms, such as hypoglycemia, skin ulcer, Cushing’s syndrome, which are called carcinoid syndrome, and are more common in pancreatic NENs. It is reported that only 15–18% of patients with NENs have carcinoid syndrome [6]. However, most of the NENs are nonfunctional, especially the bile duct NENs. It has not yet been found whether bile duct NENs can secrete certain hormones.

Most patients with bile duct and ampullary NENs presented with jaundice, accompanied by skin itching and epigastric pain. Only a few patients presented with carcinoid syndrome. Due to the lack of specific clinical symptoms, it is difficult to make a clear diagnosis only by clinical manifestations, signs, laboratory tests and imaging examinations. Therefore, bile duct and ampullary NENs are easily misdiagnosed as cholangiocarcinoma. The diagnosis of NENs needs to be confirmed by pathological examination, and immunohistochemical detection also has a certain role in assisting diagnosis. The main treatment for bile duct and ampullary NENs is surgery, and adjuvant chemotherapy and radiotherapy are also performed. However, it is still controversial whether or not chemotherapy and radiotherapy can improve the prognosis of patients.

In view of the low incidence rate and the difficulty of diagnosis, the recognition of bile duct and ampullary NENs is still very elementary, and the diagnosis and treatment are not standardized. At present, the understanding of bile duct NENs is mainly based on case reports and limited cases from a single institution, lacking multi-center large sample size and long-term follow-up data of patients. Therefore, the main purpose of our research is to explore and summarize the clinicopathological features and prognosis factors of bile duct and ampullary NENs using the Surveillance, Epidemiology, and End Results (SEER) database.

## 2. Materials and Methods

### 2.1. Data Resource

The clinicopathological and prognostic data were collected from the SEER database and extracted by SEER*Stat 8.3.8 software (http://seer.cancer.gov/seerstat/, accessed on 30 April 2021). The SEER database is the most representative large-scale cancer registry database in North America, which include 18 cancer registries’ clinical data and covers 34.6% of the U.S. population [7], and with the characteristics of relatively complete follow-up information and large sample size. The cancer information released by the SEER database is allowed to be reported publicly; therefore, the Ethics Committee of the First Affiliated Hospital of Xi’an Jiaotong University exempted the review of this study.

### 2.2. Population Selection

According to the International Classification of Disease for Oncology (3rd edition) (ICD-O-3) site record, the bile duct was defined with C22.1, C24.0-C24.9. At the same time, neuroendocrine neoplasms involve the following ICD-O-3 histological codes: 8013, 8240–8246 and 8249. Patients with missing survival information were excluded. After the final screening, we identified 340 patients who met the above criteria from 1975 to 2016.

### 2.3. Variables and Outcome

Age, gender, race, primary site, marital status, histological grade, SEER stage, TNM stage, surgery, chemotherapy and radiotherapy data were extracted from the SEER database for analysis. The overall survival (OS) was defined as the period from the date of diagnosis to death from any cause. The disease-specific survival (DSS) referred to the date of diagnosis to death due to primary tumor.

### 2.4. Statistical Analysis

Frequency tables were used to describe the baseline characteristics of patients. The Kaplan–Meier curve and the log-rank test were performed for survival analysis. Cox proportional hazards model analysis identified independent prognostic factors. Then, the nomogram was drawn according to independent survival predictors. The calibration of the nomogram was carried out by 1000 bootstrap resampling internal verification and displayed by the calibration curve. The concordance index (C-index) was used to quantitatively display the discrimination of the nomogram. The clinical utility and net benefit of the nomogram were evaluated by decision curve analysis (DCA). The SPSS (version 22.0, IBM Corporation, Armonk, NY, USA) and R software (version 4.0.3; The R Foundation for Statistical Computing, Vienna, Austria) were used to complete the calculation. Bilateral *p* < 0.05 was considered statistically significant.

## 3. Results

### 3.1. Patient Characteristics

A total of 340 patients were included in this study according to the screening criteria. As shown in Table 1, female and male patients accounted for 47.1% (160/340) and 52.9% (180/340) of all primary bile duct and ampullary NENs, respectively. Of all patients, 46.5% (158/340) were <60 years, 53.5% (158/340) were ≥60 years, and 76.5% (260/340) were White, 14.4% (49/340) were Black. In total, 21.8% (74/340) of cases were T1/T2, 17.9% (61/340) of cases were N0, 32.4% (110/340) of cases were M0, 29.4% (100/340) of tumors were well-differentiated, and 40.0% (136/340) of patients had regional disease. As for the primary site, 2.1% (7/340) of tumors were located in the intrahepatic bile duct, 20.9% (71/340) in the extrahepatic bile duct, 75.0% (255/340) in the ampulla of Vater, and 2.1% (7/340) had overlapping lesions of biliary tract/ biliary tract, NOS tumor. Among the cohort, 73.2% (249/340) of patients received surgery, 7.9% (27/340) underwent radiotherapy, and 16.8% (57/340) accepted chemotherapy. In total, 57.6% (196/340) of patients were registered as married.

### 3.2. Kaplan–Meier Curve Analysis

**Figure 1:**
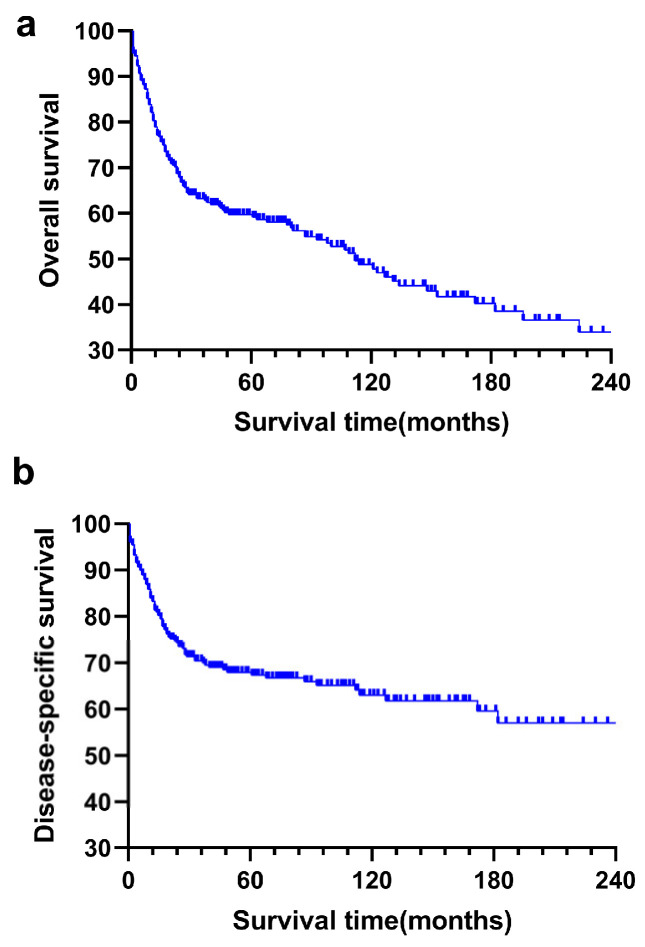
Kaplan–Meier curve analyses of the overall survival (OS, (**a**), *p* < 0.001) and disease-specific survival (DSS, (**b**), *p* < 0.001) in primary bile duct and ampullary neuroendocrine neoplasms patients.

**Figure 2:**
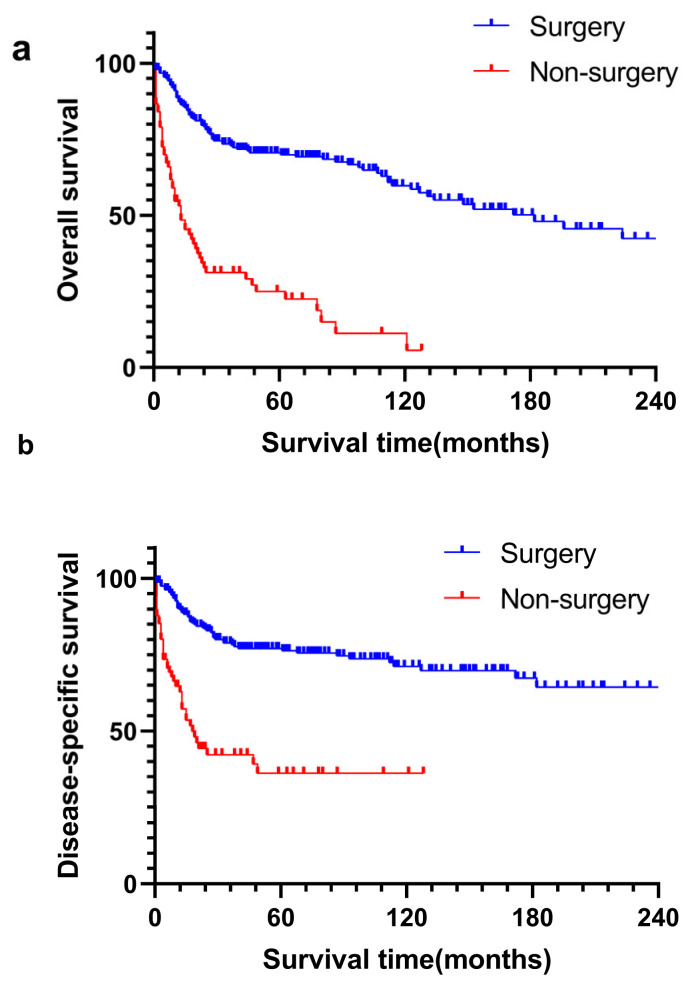
The overall survival (OS, (**a**), *p* < 0.001) and disease-specific survival (DSS, (**b**), *p* < 0.001) analyses for primary bile duct and ampullary neuroendocrine neoplasms patients in surgery and non-surgery subgroups.

During the 74.0 ± 6.5 months of median follow-up time, 149 patients were eventually reported dead, of which 102 died of the primary tumor. The median survival time was 112.0 ± 15.7 months. According to Kaplan–Meier analysis, 1-year, 3-year and 5-year of OS were 77.3%, 61.9% and 58.4% (Figure 1a), while 1-year, 3-year and 5-year of DSS were 82.7%, 69.3% and 66.9%, respectively (Figure 1b). In subgroup analysis (Figure 2), patients who underwent surgery tended to have better survival for OS [hazard ratio (HR): 4.091, 95% confidence interval (CI) = 2.562–6.532, *p* < 0.001] and DSS [hazard ratio (HR): 3.799, 95% confidence interval (CI) = 2.213–6.524, *p* < 0.001].

### 3.3. Cox Proportional Hazard Model

In order to further identify the independent risk factors affecting the prognosis, we applied the Cox proportional hazard model. The multivariable analysis results showed that age, histological grade, SEER stage and surgery were independent predictors for either OS (Table 2) or DSS (Table 3). The patients with <60 years, well-differentiated, localized and the implementation of surgery lead to better survival (*p* < 0.001).

### 3.4. Development and Validation of the Nomogram Prediction

**Figure 3:**
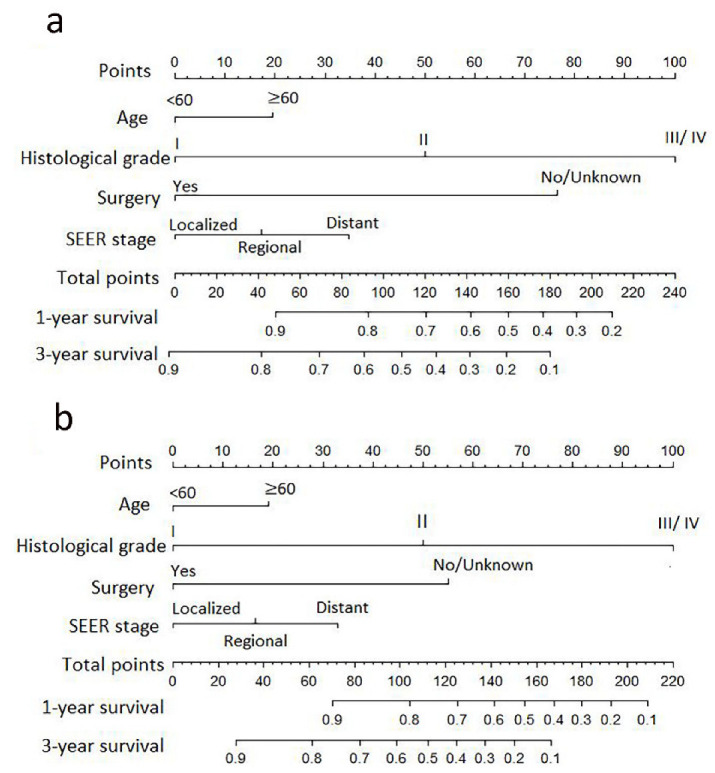
Nomogram to predict the 1-year and 3-year of the overall survival (**a**) and disease-specific survival (**b**) in primary bile duct and ampullary neuroendocrine neoplasms patients.

**Figure 4:**
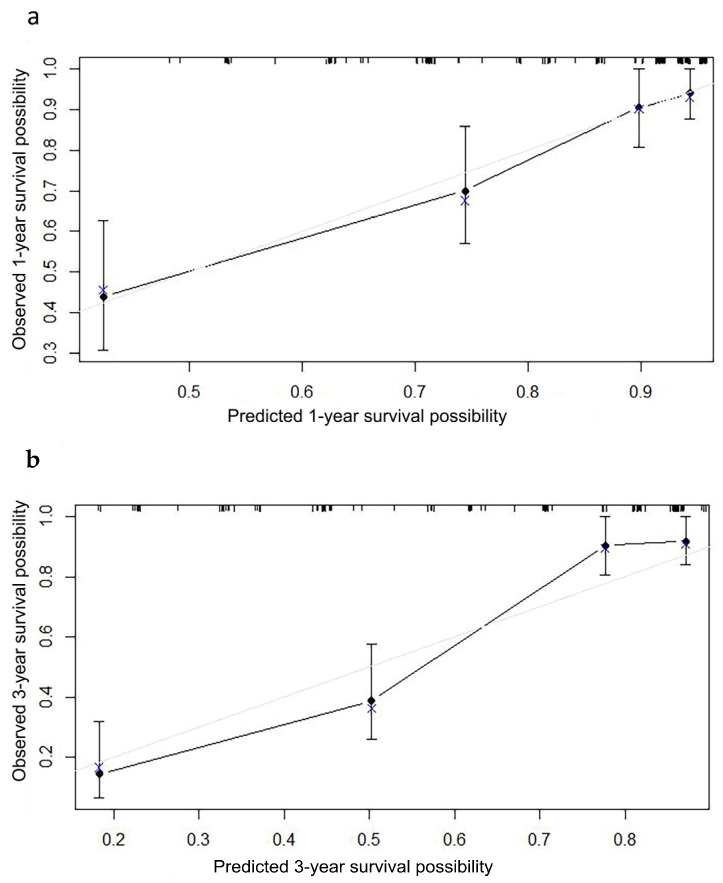
The 1000 bootstrap resampling internal verification correction for the nomogram of (**a**) 1-year overall survival, (**b**) 3-year overall survival in primary bile duct and ampullary neuroendocrine neoplasms patients.

**Figure 5:**
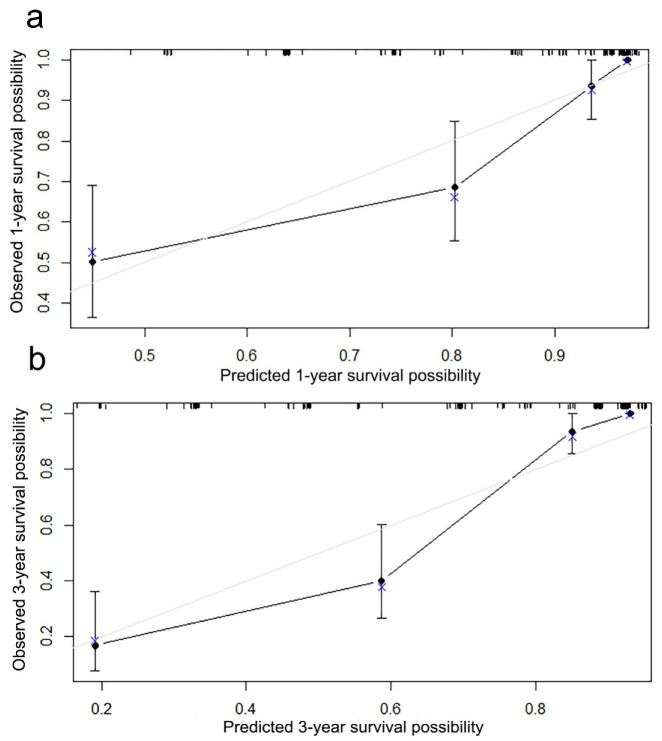
The 1000 bootstrap resampling internal verification correction for the nomogram of (**a**) 1-year disease-specific survival, (**b**) 3-year disease-specific survival in primary bile duct and ampullary neuro-endocrine neoplasms patients.

**Figure 6:**
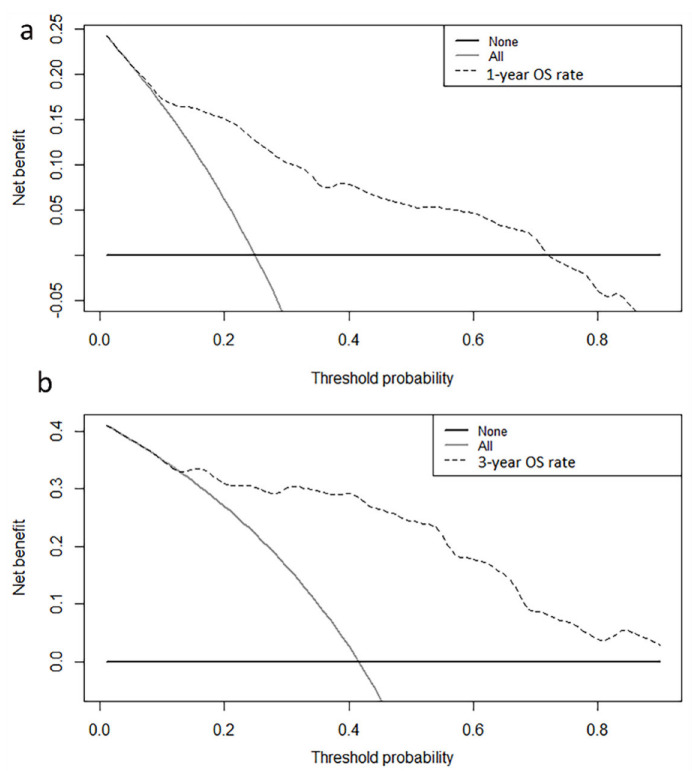
The decision curve analysis of the (**a**) 1-year overall survival rates, (**b**) 3-year overall survival rates for the training and validation cohorts.

**Figure 7:**
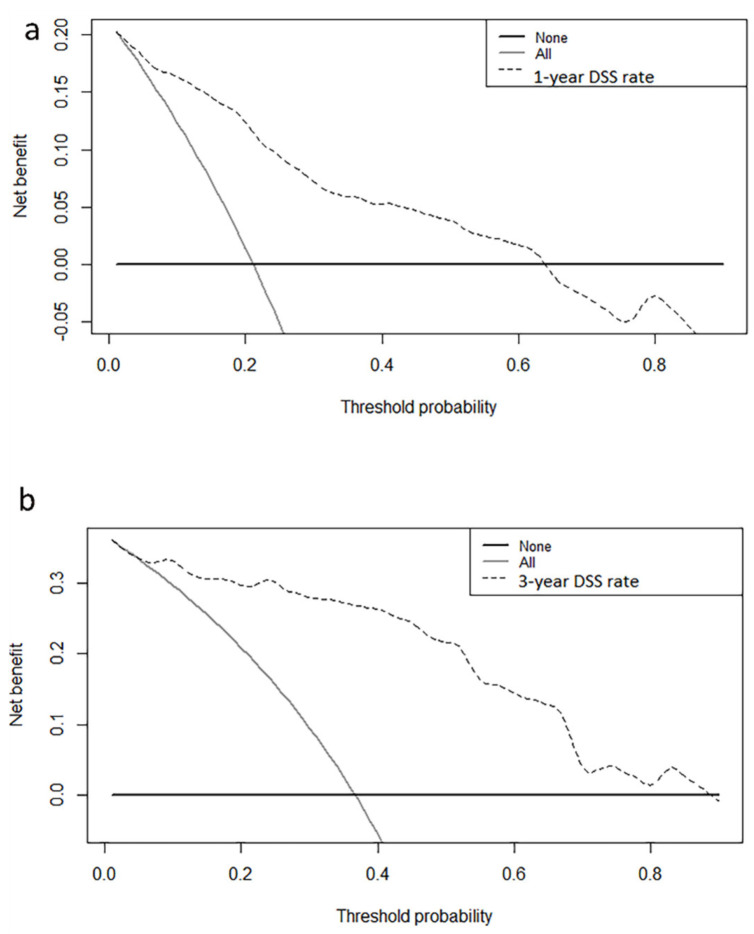
The decision curve analysis of the (**a**) 1-year disease-specific survival rates, (**b**) 3-year disease-specific survival rates for the training and validation cohorts.

A nomogram was developed to predict the OS (Figure 3a) and DSS (Figure 3b) of primary bile duct neuroendocrine neoplasms patients, according to age, histological grade, SEER stage and surgery. The predicted survival rate can be obtained by summing the scores of these four factors. In the 1000 bootstrap resampling internal verification calibration curve for 1-year and 3-year OS (Figure 4) and DSS (Figure 5), the trend in the true value and predicted value were both consistent, proving the nomogram was well calibrated. The C-index value was 0.797 (95% CI 0.741–0.853) of OS prediction, and 0.844 (95% CI 0.798–0.890) of DSS prediction, all of which proved that the nomogram had good discrimination and good prediction ability. The DCA curve showed that the 1-year and 3-year OS (Figure 6) and DSS (Figure 7) rates predicted by nomogram were better than treating all patients or not treating any patients within the threshold probability range. Therefore, the model had ideal clinical utility and net benefits.

## 4. Discussion

In the past, the definition of NENs, which were also called carcinoid syndrome, was vague. Until 2010, the World Health Organization (WHO) classified digestive system NENs into three subtypes: well differentiated NET, poorly differentiated neuroendocrine carcinoma (NEC) and mixed adenoneuroendocrine carcinoma (MANEC) according to their histological characteristics and proliferation rate [8]. The NEN was further divided into Grade 1–3 based on the mitotic number and Ki-67 index. Moreover, there is no special TNM stage for bile duct NENs, which was the same as that of cholangiocarcinoma. Scientists’ understanding of bile duct NENs needs to be further standardized and improved.

In our research, we analyzed the clinicopathological features and prognosis of bile duct and ampullary NEN patients from 1975 to 2016 by using the SEER database, included the primary sites of the intrahepatic bile duct, the extrahepatic bile duct and the ampulla of Vater. The Cox proportional hazard model confirmed that age, histological grade, SEER stage and surgery were independent prognostic factors. In addition, we constructed the nomogram to predict the survival rate, and evaluated the accuracy, discrimination and clinical benefits of the model. The results showed that our nomogram has a high predictive value. As far as we know, this is the most comprehensive and largest report for bile duct and ampullary NENs using the SEER database.

As for different primary sites of bile duct NENs, they all have a good long-term survival, and there is no statistical difference in survival rates. The incidence rate of ampullary NENs was the highest, and in a report of 120 ampullary NEN patients, the median OS was 98 months [9], which is similar to our results. For extrahepatic bile duct NENs, the existing studies are mainly case reports, and the survival time of patients varies greatly [10,11,12], which may be related to the small number of patients and different tumor grades. Intrahepatic bile duct NENs are the rarest among them. There are no large retrospective studies, and whether there exist intrahepatic bile duct NENs is still controversial [13].

Bile duct NENs have been proved to have a considerably favorable prognosis, which may be related to the fact that bile duct NENs are inert tumors with limited ability of local diffusion and metastasis, and their distant metastasis rate is also low [3,14,15]. In our study, 73.2% of patients received surgical treatment, and patients who received surgery had better OS (HR: 4.091, 95% CI = 2.562–6.532, *p* < 0.001) and DSS (HR: 3.799, 95% CI = 2.213–6.524, *p* < 0.001). Therefore, our nomogram confirms that surgical resection can be regarded as the main treatment for bile duct and ampullary NENs. Early determination of the surgical method and radical resection according to the tumor location can maximize the prognosis of patients. For patients with metastatic tumors who cannot undergo radical resection, tumor reduction surgery may also slow tumor development and improve the survival rate [16,17,18].

Our nomogram showed that the prognosis of patients who received chemotherapy was worse than that of patients who did not, which may be due to the later tumor stage or poor tumor sensitivity to chemotherapy. It has been reported that the somatostatin analog octreotide can improve the progression-free survival rate of gastrointestinal carcinoid patients [19]. However, whether it is effective in patients with bile duct NEN has not been confirmed. In recent years, gene-targeted therapy for NEN has gradually developed [20,21], and molecular therapy may become the development direction of therapy in the future. In our study population, most patients (83.2%) did not receive chemotherapy. As bile duct NEN is very rare in the clinic, little is known about its pathogenesis. In addition, it is difficult to culture bile duct NEN cells in vitro for a long time and establish an in vivo model for new drug research. Therefore, whether chemotherapy should be used, the timing of chemotherapy and the chemotherapy scheme are all challenging issues.

Inevitably, there are some shortcomings in this paper. First, this is a retrospective study and large prospective studies are needed to verify our results. Second, the SEER database has the phenomenon of data loss, and lacks data such as grade, surgical methods and chemotherapy regimens, which are also important factors affecting survival. Third, because of the low incidence rate of bile duct NEN, the number of patients is relatively small, which may lead to statistical bias. In addition, elderly patients account for a large proportion of our population, 53.5% of whom are over 60 years old. A total of 26.8% of the patients in our cohort did not receive surgery; however, the reasons (unsuitable for surgical treatment or patients refused surgical treatment) were not recorded in the database. Whether the patients included in the analysis had concomitant diseases is not shown in the database, and concomitant acute or chronic diseases may affect the survival of patients. All these factors will lead to possible population selection bias in our study. In further research, we will conduct a randomized controlled study to reduce such bias.

At present, scientists have a limited understanding of bile duct and ampullary NENs, and there are considerable difficulties in clinical diagnosis and treatment. As a special type of bile duct tumor, it deserves long-term attention, although its incidence rate is relatively low. Specific, suitable and separate criteria are required for bile duct and ampullary NEN staging and grading. How to formulate a comprehensive treatment plan and choose the best surgery, chemotherapy and radiotherapy methods all need large clinical studies to provide evidence. Furthermore, basic biological research should be conducted to elucidate the pathogenesis and biological behavior of bile duct and ampullary NENs.

## 5. Conclusions

The age, histological grade, SEER stage and surgery were identified as independent prognostic variables for OS and DSS. After verification, nomogram has good predictive ability and clinical application value.

## Figures and Tables

**Table 1:** Clinical characteristics of 340 patients with primary bile duct and ampullary neuroendocrine neoplasms.

Characteristics	Number of Patients (*N* = 340)	Percentage (%)
Sex		
Female	160	47.1
Male	180	52.9
Age at diagnosis (yr)		
<60	158	46.5
≥60	182	53.5
Race		
White	260	76.5
Black	49	14.4
Other	31	9.1
Primary site		
Intrahepatic bile duct	7	2.1
Extrahepatic bile duct	71	20.9
Ampulla of Vater	255	75.0
Overlapping lesions of biliary tract/	7	2.1
Biliary tract, NOS		
T stage		
T1/T2	74	21.8
T3/T4	39	11.5
Unknown	227	66.8
N stage		
N0	61	17.9
N1	60	17.6
Unknown	219	64.4
M stage		
M0	110	32.4
M1	25	7.4
Unknown	205	60.3
Histological grade		
Well differentiated, I	100	29.4
Moderately differentiated, II	37	10.9
Poorly differentiated, III/	68	20.0
Undifferentiated, IV		
Unknown	135	39.7
SEER stage		
Localized	86	25.3
Regional	136	40.0
Distant	51	15.0
Unknown	67	19.7
Surgery		
Yes	249	73.2
No/Unknown	91	26.8
Radiotherapy		
Yes	27	7.9
No/Unknown	313	92.1
Chemotherapy		
Yes	57	16.8
No/Unknown	283	83.2
Marriage status		
Married	196	57.6
Unmarried/DSW	144	42.4

DSW: divorced and separated and widowed.

**Table 2:** Univariable and multivariable Cox proportional hazard model analysis of overall survival in patients with primary bile duct and ampullary neuroendocrine neoplasms.

Characteristics	Univariable Analysis			Multivariable Analysis		
	HR	95% CI	*p*	HR	95% CI	*p*
Sex			0.612			
Female	Reference					
Male	0.920	0.667–1.270				
Age at diagnosis (yr)			<0.001			<0.001
<60	Reference			Reference		
≥60	3.570	2.470–5.159		2.397	1.627–3.532	
Race			0.274			
White	Reference					
Black	0.667	0.401–1.109	0.118			
Other	1.064	0.600–1.886	0.833			
Primary site			0.264			
Intrahepatic bile duct	Reference					
Extrahepatic bile duct	0.451	0.175–1.160	0.098			
Ampulla of Vater	0.460	0.187–1.129	0.090			
Overlapping lesions of biliary tract	0.789	0.211–2.947	0.725			
Biliary tract, NOS						
T stage			0.002			
T1/T2	Reference					
T3/T4	3.049	1.653–5.623	<0.001			
Unknown	1.675	1.007–2.786	0.047			
N stage			0.382			
N0	Reference					
N1	1.511	0.843–2.707	0.166			
Unknown	1.272	0.779–2.075	0.336			
M stage			<0.001			
M0	Reference					
M1	4.455	2.584–7.682	<0.001			
Unknown	1.177	0.784–1.767	0.432			
Histological grade			<0.001			<0.001
Well differentiated, I	Reference			Reference		
Moderately differentiated, II	1.381	0.666–2.864	0.386	1.588	0.759–3.323	0.220
Poorly differentiated, III/	5.953	3.552–9.977	0.000	4.140	2.425–7.068	<0.001
Undifferentiated, IV	1.762	1.072–2.899	0.026			
Unknown				1.517	0.921–2.501	0.102
SEER stage			<0.001			<0.001
Localized	Reference			Reference		
Regional	1.327	0.866–2.033	0.194	1.314	0.839–2.057	0.233
Distant	5.107	3.206–8.133	<0.001	2.100	1.26–3.501	0.004
Unknown	1.050	0.560–1.969	0.878	0.642	0.336–1.226	0.179
Surgery			<0.001			<0.001
Yes	Reference			Reference		
No/Unknown	4.628	3.279–6.532		3.594	2.420–5.337	
Radiotherapy			0.300			
Yes	Reference					
No/Unknown	0.754	0.442–1.286				
Chemotherapy			<0.001			
Yes	Reference					
No/Unknown	0.485	0.331–0.712				
Marriage status			0.117			
Married	Reference					
Unmarried/DSW	1.294	0.938–1.785				

DSW: divorced and separated and widowed.

**Table 3:** Univariable and multivariable Cox proportional hazard model analysis of disease-specific survival in patients with primary bile duct and ampullary neuroendocrine neoplasms.

Characteristics	Univariable Analysis			Multivariable Analysis		
	HR	95% CI	*p*	HR	95% CI	*p*
Sex			0.967			
Female	Reference					
Male	1.008	0.682–1.490				
Age at diagnosis (yr)			<0.001			<0.001
<60	Reference			Reference		
≥60	3.712	2.351–5.861		2.369	1.463–3.835	
Race			0.039			
White	Reference					
Black	0.384	0.178–0.830	0.015			
Other	1.034	0.520–2.055	0.925			
Primary site			0.095			
Intrahepatic bile duct	Reference					
Extrahepatic bile duct	0.465	0.162–1.340	0.156			
Ampulla of Vater	0.376	0.137–1.032	0.058			
Overlapping lesions of biliary tract	0.896	0.224–3.591	0.877			
Biliary tract, NOS						
T stage			0.003			
T1/T2	Reference					
T3/T4	3.222	1.626–6.384	0.001			
Unknown	1.572	0.879–2.811	0.127			
N stage			0.282			
N0	Reference					
N1	1.718	0.873–3.379	0.117			
Unknown	1.367	0.764–2.446	0.293			
M stage			<0.001			
M0	Reference					
M1	5.716	3.119–10.478	<0.001			
Unknown	1.288	0.793–2.090	0.307			
Histological grade			<0.001			<0.001
Well differentiated, I	Reference			Reference		
Moderately differentiated, II	2.373	0.964–5.840	0.060	2.375	0.933–6.043	0.070
Poorly differentiated, III	11.183	5.614–22.277	<0.001	6.683	3.290–13.576	<0.001
Undifferentiated, IV						
Unknown	2.214	1.092–4.487	0.027	2.027	0.944–4.136	0.052
SEER stage			<0.001			<0.001
Localized	Reference			Reference		
Regional	2.557	1.381–4.734	0.003	2.610	1.316–5.175	0.006
Distant	9.289	4.868–17.724	<0.001	4.245	2.049–8.794	<0.001
Unknown	1.446	0.597–3.502	0.414	0.900	0.362–2.233	0.819
Surgery			<0.001			<0.001
Yes	Reference			Reference		
No/Unknown	4.213	2.812–6.313		3.243	1.994–5.275	
Radiotherapy			0.079			
Yes	Reference					
No/Unknown	0.593	0.331–1.062				
Chemotherapy			<0.001			
Yes	Reference					
No/Unknown	0.322	0.212–0.489				
Marriage status			0.669			
Married	Reference					
Unmarried/DSW	0.917	0.616–1.365				

DSW: divorced and separated and widowed.

## Data Availability

The datasets generated during and/or analyzed during the current study are available in the SEER repository (http://seer.cancer.gov/seerstat/, accessed on 30 April 2021).
